# Optimizing treatment with tumour necrosis factor inhibitors in rheumatoid arthritis—a proof of principle and exploratory trial: is dose tapering practical in good responders?

**DOI:** 10.1093/rheumatology/kex315

**Published:** 2017-08-17

**Authors:** Fowzia Ibrahim, Beatriz Lorente-Cánovas, Caroline J Doré, Ailsa Bosworth, Margaret H Ma, James B Galloway, Andrew P Cope, Ira Pande, David Walker, David L Scott

**Affiliations:** 1 Academic Department of Rheumatology, Division of Immunology, Infection and Inflammatory Disease, Faculty of Life Sciences and Medicine, King's College London; 2 Comprehensive Clinical Trials Unit, University College London, London; 3 National Rheumatoid Arthritis Society, Maidenhead; 4 Rheumatology Department, Nottingham University Hospitals NHS Trust, Nottingham; 5 Musculoskeletal Services, Freeman Hospital, Newcastle, UK

**Keywords:** TNF, RA, biologics, tapering, interruption treatment, flare

## Abstract

**Objectives:**

RA patients receiving TNF inhibitors (TNFi) usually maintain their initial doses. The aim of the Optimizing Treatment with Tumour Necrosis Factor Inhibitors in Rheumatoid Arthritis trial was to evaluate whether tapering TNFi doses causes loss of clinical response.

**Methods:**

We enrolled RA patients receiving etanercept or adalimumab and a DMARD with DAS28 under 3.2 for over 3 months. Initially (months 0–6) patients were randomized to control (constant TNFi) or two experimental groups (tapering TNFi by 33 or 66%). Subsequently (months 6–12) control subjects were randomized to taper TNFi by 33 or 66%. Disease flares (DAS28 increasing ⩾0.6 with at least one additional swollen joint) were the primary outcome.

**Results:**

Two hundred and forty-four patients were screened, 103 randomized and 97 treated. In months 0–6 there were 8/50 (16%) flares in controls, 3/26 (12%) with 33% tapering and 6/21 (29%) with 66% tapering. Multivariate Cox analysis showed time to flare was unchanged with 33% tapering but was reduced with 66% tapering compared with controls (adjusted hazard ratio 2.81, 95% CI: 0.99, 7.94; P = 0.051). Analysing all tapered patients after controls were re-randomized (months 6–12) showed differences between groups: there were 6/48 (13%) flares with 33% tapering and 14/39 (36%) with 66% tapering. Multivariate Cox analysis showed 66% tapering reduced time to flare (adjusted hazard ratio 3.47, 95% CI: 1.26, 9.58; P = 0.016).

**Conclusion:**

Tapering TNFi by 33% has no impact on disease flares and appears practical in patients in sustained remission and low disease activity states.

**Trail registration:**

EudraCT, https://www.clinicaltrialsregister.eu, 2010-020738-24; ISRCTN registry, https://www.isrctn.com, 28955701

Rheumatology key messages

- Tapering TNF inhibitors in RA patients by 33% over 6 months did not reduce time to disease flare.
- By 12 months, 45% of RA patients who tapered TNF inhibitor treatment were able to stop it entirely.

## Introduction

Trials and observational studies underpinning the regulatory approval of TNF inhibitors (TNFi) for treating RA patients focus on initial efficacy and long-term safety [1–3]. What to do after achieving disease control is another question. Currently doses of TNFi effective in inducing responses in active RA are continued to maintain control, despite limited evidence they are needed. Maintaining disease control maintained with lower doses of TNFi should increase their cost-effectiveness.

The Optimizing Treatment with TNFi in RA (OPTTIRA) trial was designed when there was limited information about tapering TNF inhibitor in RA patients with good treatment responses. Subsequently several observational studies and trials evaluated tapering. Systematic reviews of reports before 2015 [4–7] found evidence supporting TNF tapering. The Cochrane systematic review, which focused exclusively on trials [7], found tapering gave similar outcomes to continuing standard doses of adalimumab and etanercept; complete discontinuation increased flares. Another seven trials [8–14] were published after these systematic reviews were completed. One trial [12] concluded disease activity guided adalimumab or etanercept dose reductions were non-inferior to usual care for major flares; dose reduction or stopping was possible in two-thirds of patients. Overall the evidence suggests tapering does not substantially increase flares; stopping completely may do so. Recent observational studies support dose reduction [15–17]. Reviews by Schett *et al.* [18] and Edwards *et al.* [19] highlighted the importance of TNF inhibitor tapering and minimizing other long-term DMARDs. However, there remain uncertainties about which patients should have their TNFi tapered and whether all tapering regimens are similar.

OPTTIRA is a randomized trial evaluating two tapering regimens in RA. It recruited patients showing EULAR good responses to TNFi [20] and compared tapering with continuing standard doses. Tapering regimens reduced doses to one-third and two-thirds initial response induction doses of adalimumab or etanercept over 6 months. OPTTIRA also examined subsequently stopping TNFi completely. It used time to flare to assess the effects of TNF inhibitor tapering. Flares occurred when the DAS28 joints was over 3.2 and increased by 0.6 or more.

## Methods

### Design

OPTTIRA was an open label, 6-month multicentre proof of principle trial with a subsequent 6-month exploratory phase for patients who completed the initial trial. OPTTIRA enrolled RA patients achieving good responses [20] with low disease activity or remission taking standard TNFi doses and receiving one or more DMARDs.

### Participants

Patients receiving TNFi had met existing English criteria from the National Institute for Health and Clinical Excellence for these agents. The criteria have changed with time; they included failing to respond to MTX and another DMARD [21]. These criteria meant all patients had established RA. Patients had also achieved sustained good responses with DAS28 scores of ⩽3.2 without increases of > 0.6 during the previous 3 months.

### Interventions

Patients were taking etanercept or adalimumab; their existing TNFi were the trial investigational medicinal products.

The proof of principle trial (months 0–6) compared three groups: experimental group 1: TNF inhibitor tapered by 33% initial dose; experimental group 2: TNF inhibitor tapered by 66% initial dose; control group: continued standard doses. Supplementary Tables S1 and S2, available at *Rheumatology* Online, show reducing etanercept and adalimumab dosing schedules.

In the exploratory phase (months 7–12) patients in experimental groups increased times between injections until they stopped. Patients in the control group were further randomized into two groups: control group A had TNFi tapered by 33% initial dose; control group B had TNFi tapered by 66% initial dose. Supplementary Tables S3 and S4, available at *Rheumatology* Online, show the dosing schedules. The tapering schedules reflected standard dosing regimens related to the half-lives of the drugs.

### Primary outcome

The primary outcome was time to first flare, defined as an increase in DAS28 scores ⩾0.6 resulting in a DAS28 >3.2 together with an increase in the swollen joint count; both had to be present on two occasions at least 1 week apart. An increase in DAS28 score ⩾1.2 resulting in DAS28 >3.2 was defined as flare irrespective of changes in swollen joints. These criteria reflect the subsequently developed DAS28 flare definitions proposed by OMERACT [22] and supported by the Cochrane group [7]. Patients were assessed 3 monthly and telephoned by their Research Nurse monthly to check their disease control. Patients who considered they were experiencing a flare were seen urgently (within 2 weeks).

### Secondary outcomes

HAQ, EuroQol 5-dimension scale (EQ5D-3L), Medical Outcomes Study 36-Item Short Form Health Survey (SF-36) and Functional Assessment of Chronic Illness Therapy were assessed at 0, 3, 6 and 12 months. X-rays of the hands (including wrists) and feet were taken at 0, 6 and 12 months with digitized X-rays read by an experienced observer (D.L.S.) blinded to treatment using modified Larsen scores. Every 3 months disease activity assessments recorded tender and swollen joint counts (28 joints), ESR, patients’ global assessments of disease activity (100 mm visual analogue scale) and DAS for 28 joints together with details of medication and adverse effects. An anonymized electronic data capture system collected clinical data except X-rays (http://www.medscinet.net).

### Sample size

The TEMPO trial showed 15% of patients on etanercept with MTX withdrew annually (flares and other problems) [23, 24]. The BSR Biologics Register [25] showed 12% of patients withdraw annually from TNFi. These results suggested 12–15% of patients taking TNFi flared annually; during the 6 months proof of principle trial the likelihood of flare was 7.5%. Studying 30 patients in one tapering group would show a significant difference at the 5% level with 80% power if over 42% of patients flared; such a difference would mean tapering was not clinically useful. Therefore a proof of principle study of 30 patients in each group should provide sufficient information to reject the concept if it is clearly ineffective, as well as giving enough data to design larger trials if needed. We allowed for 10% of patients not continuing in the trial and therefore aimed to recruit 33 patients in each group with a total study size of 99 patients.

The randomization algorithm considered patients in both proof of principle trial and the exploratory study taking different biologics. Consequently the randomization ratio generated was 1:1:2; we recruited twice as many to the control group than the initial tapering groups in the proof of principle trial.

### Randomization

Potentially eligible patients were screened and reasons for non-entry recorded. The electronic data capture system (MedSciNet) randomized patients using minimization into experimental and control groups, stratified by TNF inhibitor (etanercept or adalimumab) using randomly permuted blocks. Recruiting staff were blinded to allocation sequences.

### Blinding

OPTTIRA was unblinded for assessors because it used treatment-specific algorithms with the adjustment of multiple dosing intervals of the drugs. Disability and quality of life were assessed by patients and X-ray reading was blinded.

### Statistical methods

Randomized patients accepting their allocated treatment were analysed on an intention-to-treat basis. Data management and analyses used Stata (version 14.0, StataCorp, College Station, TX, USA). Baseline characteristics were summarized by randomization group as means and standard deviations (normally distributed variables), medians and interquartile ranges (non-normally distributed variables) and frequencies and percentages (categorical variables). Serious adverse event rates in each arm were compared with controls.

Separate analysis was performed for the proof of principle phase (0–6 months) and the exploratory phase (6–12 months). The primary outcome was time to flare (months) for patients randomized to control, 33% taper or 66% taper, defined as the time from study entry to the first flare. Patients without flares, who withdrew or were lost to follow-up, were censored at the time of their last visit. Eight out of 97 patients were lost to follow-up (supplementary Fig. S1, available at *Rheumatology* Online). Kaplan–Meier curves and the log-rank test compared randomized groups. Survival analysis analysed time to flare. The validity of the assumption of proportionality required for Cox regression was investigated graphically (using Nelson–Aalen plots) as well as using Schoenfeld residuals in the final Cox models.

Secondary outcomes were analysed using mixed models to estimate treatment effects including baseline values as covariates. Working correlation matrices were unstructured, which is not unduly restrictive given that measurements are only taken at three time points. The sandwich estimator of covariance matrix was used in order to obtain appropriate (consistent) estimates of precision. All P-values were two sided.

### Ethical review

The North West London Research Ethics Committee approved OPTTIRA (REC Ref: 10/H0720/69). All enrolled patients gave written informed consent. The trial was registered with the UK Clinical Research Network and other relevant organizations (EudraCT number: 2010-020738-24; ISRCTN: 28955701).

## Results

### Patients and treatments

#### Recruitment

Between April 2011 and June 2013, 244 patients were screened, 103 randomized and 97 accepted their allocated treatment (supplementary Fig. S1, available at *Rheumatology* Online).

#### Treatment at baseline

All patients received disease modifying drugs: 82 had DMARD monotherapy; 15 received combination DMARDs. The DMARDs comprised MTX (81 patients), HCQ (18), SSZ (11) and LEF (4). All patients received etanercept (43 patients) or adalimumab (54). Two patients were taking prednisolone. The mean baseline treatment duration was 5.8 years (range 0.5–15.1 years).

#### Proof of principle phase

Of the patients, 50/97 were randomized to continue biologic at unchanged dosages, 26/97 to taper biologics by 33% and 21/97 to taper by 66%; 74/97 patients completed 6 months’ treatment, 8/97 were lost to follow-up and 15/97 discontinued tapering but were followed up; 13/97 patients stopped treatment for flares, 1/97 stopped for drug toxicity and 9/97 stopped for other reasons including 1/97 for disease progression and 5/97 at the patients’ own request.

#### Exploratory phase

Of the patients randomized to continue biologics at unchanged doses who completed the 6-month proof of principle trial, 40/50 were re-randomized in the exploratory phase: 22/40 tapered by 33% and 18/40 tapered by 66%; 32/40 patients completed 6 months’ treatment and 8/40 patients discontinued tapering but were followed up; 5/40 stopped treatment for flares, 2/40 had disease progression and 1/40 stopped at the patients’ own request.

The 34 patients who had tapered for 6 months and continued on the treatment schedule in the proof of principle trial tapered further until their biologics were completely stopped by 6 months; 21/34 completed 6 months’ treatment, 2/34 were lost to follow-up and 11/34 discontinued tapering but were followed up. All 13/34 patients stopping treatment did so because of flares.

#### Baseline data and numbers analysed

Demographic and disease assessments were similar in all patient groups (Table 1). The patients had established RA (median disease duration 11.3 years) with low DAS28-ESR (median 2.0) and low HAQ scores (median 0.50). All treated patients were analysed in the proof of principle trial.

**Table 1:** Baseline demographic and clinical characteristics

	33% tapering	66% tapering	Controls	Total
	n = 26	n = 21	n = 50	n = 97
Demographic variables				
Age, mean (s.d.), years	59 (11)	58 (9)	56 (12)	57 (11)
Height, mean (s.d.), m	1.66 (0.08)	1.67 (0.08)	1.66 (0.09)	1.66 (0.08)
Weight, mean (s.d.), kg	74.8 (15.6)	70.1 (14.4)	74.3 (16.1)	73.5 (15.5)
BMI, kg/m^2^	26.9 (21.9–31.6)	24.5 (22.6–27.8)	25.3 (23.1–29.7)	25.4 (22.6–29.4)
Disease duration, years	11.2 (6.2–19.0)	10.6 (7.3–15.9)	11.9 (7.3–16.7)	11.3 (7.3–16.7)
Female gender, n (%)	19 (73)	15 (71)	38 (76)	72 (74)
Smoking status, n (%)				
Ex	9 (43)	7 (41)	16 (35)	32 (38)
Current	2 (9)	3 (18)	7 (15)	12 (14)
Clinical variables				
Tender joint counts, 28 joints	0 (0–0)	0 (0–0)	0 (0–1.00)	0 (0–1.00)
Swollen joint counts, 28 joints	0 (0–0)	0 (0–0)	0 (0–0)	0 (0–0)
Tender joint counts, 68 joints	0 (0–1.00)	0 (0–2.00)	0 (0–2.00)	0 (0–2.00)
Swollen joint counts, 66 joints	0 (0–0)	0 (0–0)	0 (0–0)	0 (0–0)
ESR, mm/h	6 (4–24)	8 (4–19)	9 (5–20)	8 (5–19)
CRP, mg/l	5 (2–6)	4 (2–5)	5 (2–7)	5 (2–6)
Assessor global rating, mm	3 (0–10)	4 (1–10)	3 (1–9)	3 (1–10)
Patient global assessment, mm	9 (1–15)	4 (1–16)	5 (2–16)	5 (1–16)
DAS28 ESR	1.7 (1.1–2.6)	1.9 (1.3–2.6)	2.1 (1.4–2.6)	2.0 (1.3–2.6)
DAS28 CRP	2.3 (2.0–2.5)	2.2 (1.7–2.5)	2.1 (1.9–2.5)	2.2 (1.9–2.5)
HAQ	0.75 (0.13–1.38)	0.38 (0.0–0.88)	0.50 (0.13–1.50)	0.50 (0.13–1.38)
EQ5D-3L	0.79 (0.66–1.00)	0.80 (0.69–1.00)	0.74 (0.59–1.00)	0.76 (0.66–1.00)
Pain visual analogue score, mm	6 (1–10)	8 (1–19)	5 (0–25)	5 (1–19)
FACIT Fatigue Scale	40 (31– 45)	41 (35–46)	42 (36–46)	41 (35–46)
Larsen score	33 (12–76)	34 (17–63)	66 (29–89)	51 (16–82)
SF-36				
PCS	43 (38–51)	46 (37–51)	44 (32–52)	45 (34–52)
MCS	51 (43–58)	57 (48–60)	58 (52–61)	57 (49–60)

Values shown as median (interquartile range) unless otherwise stated. EQ5D-3L: EuroQol 5-dimension scale; FACIT: Functional Assessment of Chronic Illness Therapy; MCS: mental health summary score; PCS: physical health summary score; SF-36: 36-Item Short Form Health Survey.

### Primary outcome

#### Proof of principle phase

**Fig. 1:**
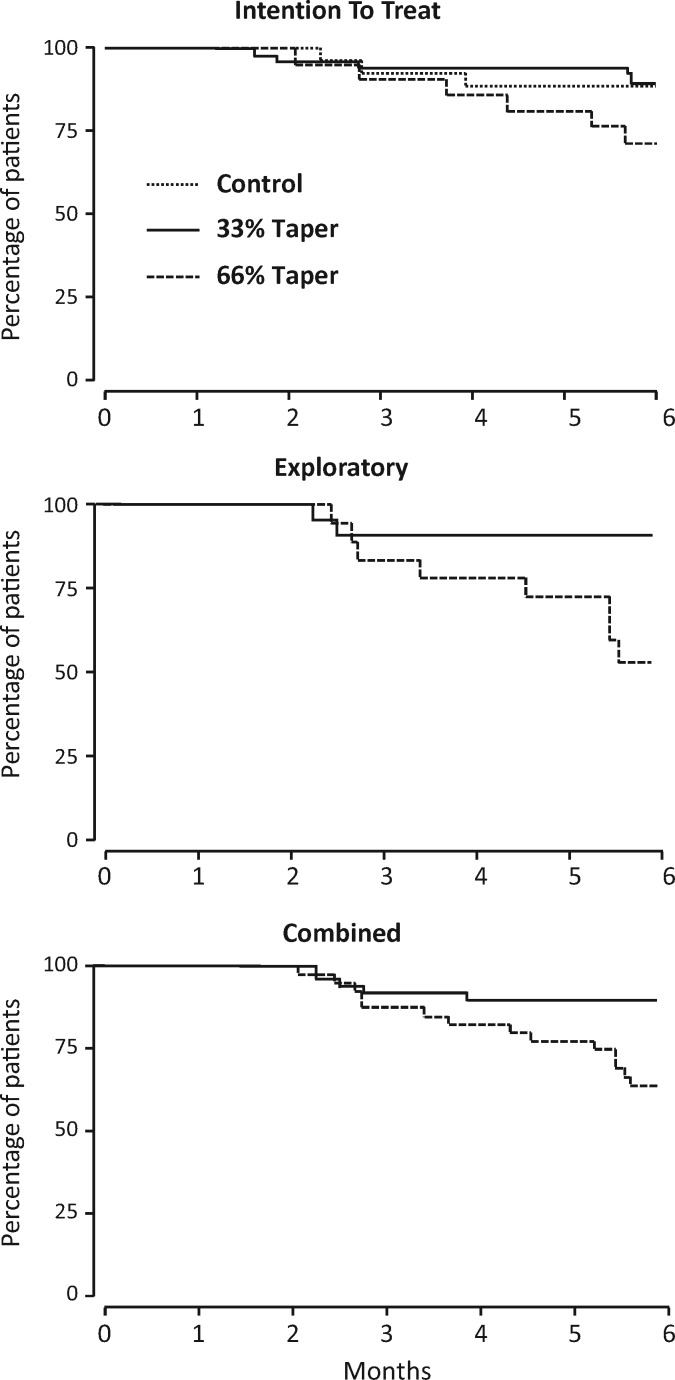
Kaplan–Meier curves for intention to treat analysis, exploratory and combined tapering groups

Seventeen patients flared comprising 8/50 (16%) control patients, 3/26 (12%) patients tapering by 33% and 6/21 (29%) patients tapering by 66% (Table 2). Univariate and multivariate Cox analysis of the intention to treat population (Table 2) showed no evidence 33% tapering reduced time to flare (adjusted hazard ratio 0.87, 95% CI: 0.22, 3.88; P = 0.835), but 66% tapering significantly reduced time to flare compared with controls (adjusted hazard ratio 2.81, 95% CI: 0.99, 7.94; P = 0.051). Figure 1 shows Kaplan–Meier survival curves comparing groups.

**Table 2:** Flare rates and univariate and multivariate cox analyses in intention to treat analysis and exploratory studies

		Flares, n (%)	Unadjusted		Adjusted^a^	
			HR (95% CI)	P-value	HR (95% CI)	P-value
Intention to treat analysis (n = 97)	Control	8/50 (16)	Reference		Reference	
	33% tapering	3/26 (12)	0.90 (0.23, 3.48)	0.873	0.87 (0.22, 3.88)	0.835
	66% tapering	6/21 (29)	2.52 (0.85, 7.48)	0.097	2.81 (0.99, 7.94)	0.051
Exploratory study (n = 40)	33% tapering	3/22 (14)	Reference		Reference	
	66% tapering	8/18 (44)	4.16 (1.08, 15.99)	0.038	5.10 (1.18, 21.95)	0.029
Combined tapering (n = 87)	33% tapering	6/48 (13)	Reference		Reference	
	66% tapering	14/39 (36)	3.29(1.26, 8.63)	0.015	3.47(1.26, 9.58)	0.016

a The multivariate model was adjusted for age at registration, gender and disease duration.

#### Exploratory phase—re-randomized patients

Eleven patients flared: 3/22 (14%) patients tapering by 33% and 8/18 (44%) tapering by 66% (Table 2). Univariate and multivariate Cox analysis showed 66% tapering significantly increased the risk of flare compared with 33% tapering (adjusted hazard ratio 5.10, 95% CI: 1.81, 21.95; P = 0.029).

##### Combined tapering groups

Results from re-randomized initial controls were combined with the tapering groups in the proof of principle trial to provide a detailed comparison of time to flare in the two tapering regimens over 6 months. Six of forty-eight (13%) patients tapering by 33% flared compared with 14/39 (36%) patients tapering by 66% (Table 2). Multivariate Cox analysis showed 66% tapering significantly increased the risk of flare compared with 33% tapering (adjusted hazard ratio 3.47, 95% CI: 1.26, 9.58; P = 0.016).

### Secondary outcomes in proof of principle trial

Longitudinal analysis of changes within the first 6 months in all treated patients (Table 3) showed little evidence that 33% tapering had any impact on clinical or functional assessments. However, 66% tapering led to significant worsening in tender and swollen joint counts, C-reactive protein levels and ESR and DAS28 ESR but not DAS28 CRP scores. An interesting finding is that patient related outcome measures including patient global score, HAQ, EQ5D-3L, visual analogue scale (VAS) pain and functional assessment of chronic illness therapy fatigue scores were unaffected by tapering, including tapering by 66%. Assessor global scores and Larsen scores also did not differ between groups. Details of secondary outcomes are shown in supplementary Table S5, available at *Rheumatology* Online.

**Table 3:** Secondary outcomes for the effect of randomized treatment arm in longitudinal analysis for first 6 months

Outcome	33% tapering				66% tapering			
	Unadjusted coefficient	P-value	Adjusted coefficient^a^	P-value	Unadjusted coefficient	P-value	Adjusted coefficient^a^	P-value
	(95% CI)		(95% CI)		(95% CI)		(95% CI)	
DAS28-ESR	0.13 (−0.20, 0.47)	0.438	0.06 (−0.25, 0.38)	0.690	0.44 (0.07, 0.80)	<0.02	0.36 (0.02, 0.70)	<0.05
DAS28-CRP	−0.05 (−0.27, 0.18)	0.671	−0.05 (−0.27, 0.18)	0.685	0.17 (−0.08, 0.41)	0.188	0.18 (−0.07, 0.42)	0.163
Tender joint counts	0.07 (−0.76, 0.91)	0.864	0.05 (−0.78, 0.88)	0.911	1.34 (0.42, 2.25)	<0.01	1.32 (0.42, 2.22)	<0.01
Swollen joint counts	−0.17 (−0.71, 0.37)	0.530	−0.21 (−0.74, 0.32)	0.438	0.97 (0.39, 1.56)	0.001	0.88 (0.30, 1.46)	<0.01
ESR	−0.97 (−5.06, 3.11)	0.641	−1.07 (−4.15, 2.00)	0.494	−3.47 (−7.89, 0.96)	0.124	−3.87 (−7.21, −0.53)	<0.05
CRP	−2.60 (−5.50, 0.30)	0.079	−2.86 (−5.67, −0.05)	0.050	−4.34 (−7.49, −1.18)	0.01	−4.28 (−7.34, −1.21)	<0.01
Assessor global	0.86 (−5.15, 6.86)	0.779	0.72 (−5.30, 6.73)	0.815	5.23 (−1.27, 11.73)	0.115	5.11 (−1.40, 11.63)	0.124
Patient global	2.64 (−3.32, 8.59)	0.385	3.00 (−2.73, 8.74)	0.305	4.82 (−1.62, 11.27)	0.142	5.34 (−0.87, 11.55)	0.092
HAQ	0.04 (−0.10, 0.17)	0.591	0.03 (−0.10, 0.15)	0.690	0.15 (0.01, 0.30)	<0.05	0.13 (−0.01, 0.27)	0.071
EQ5D-3L	−0.04 (−0.12, 0.03)	0.249	−0.03 (−0.10, 0.04)	0.402	−0.05 (−0.13, 0.03)	0.248	−0.04 (−0.11,0.04)	0.326
VAS pain	6.62 (−1.83, 15.06)	0.125	5.10 (−2.49, 12.69)	0.188	3.85 (−5.30, 12.99)	0.410	0.79 (−7.55, 9.13)	0.853
FACIT fatigue	−0.35 (−2.93, 2.24)	0.793	−0.58 (−3.01, 1.85)	0.642	1.41 (−1.38, 4.20)	0.321	1.03 (−1.60, 3.65)	0.444
Larsen scores	−0.21 (−1.69, 1.27)	0.780	0.13 (−1.39, 1.64)	0.665	0.35 (−1.25, 1.96)	0.867	0.63 (−0.98, 2.25)	0.437

a The model was adjusted for age at registration, gender and disease duration. EQ5D-3L: EuroQol 5-dimension scale; FACIT: Functional Assessment of Chronic Illness Therapy; VAS: visual analogue scale.

### Impact of flares in proof of principle phase

The effects of flares, irrespective of treatment group, on 6-month outcomes were assessed in linear regression models adjusted for age, gender and disease duration. DAS28 scores were higher in patients who flared (0.85, 95% CI: 0.44, 1.26; P < 0.001) and EQ5D scores were lower (−0.12, 95% CI: −0.23, −0.02; P = 0.022), but HAQ scores were unaffected (0.92, 95% CI: −0.19, 0.22; P = 0.87).

### Discontinuing TNF inhibitor

Thirty-four patients who tapered biologics in the proof of principle trial (21 with 33% tapering; 13 with 66% tapering) further tapered treatment until they stopped TNF inhibitor over 6 months in the exploratory study. Thirteen of thirty-four (38%) flared and 21/34 (62%) did not flare. Consequently 21/47 (45%) patients who started tapering in the proof of principle trial stopped TNF inhibitor by 12 months; 21/47 (45%) had flared and 5/47 (11%) had stopped tapering for other reasons. The 21 patients who stopped TNF inhibitor without flaring had initial DAS28 score of 1.51 (95% CI: 1.14, 2.65) and 12 month DAS28 scores of 2.27 (95% CI: 1.71, 3.98). Their initial HAQ was 0.65 (95% CI: 0.33, 0.99) and the 12 month HAQ 0.75 (95% CI: 0.42, 1.17).

### Flares in patient subgroups

The flare rate for patients receiving adalimumab, 18/54 (33%), was significantly lower (χ^2^ = 3.99, degrees of freedom (DF) = 1, P = 0.050) than for patients receiving etanercept, 23/43 (53%).

At baseline 74 patients were in remission and had DAS28 scores under 2.6; 27/74 (36%) of these patients flared. There were 23 patients with low disease activity (DAS28 score 2.6–3.2) and 14/23 (61%) of these flared. The risk of flare was therefore significantly higher for patients with low disease activity at baseline (χ^2^ = 4.3, DF = 1, P = 0.039) than for those in remission.

### Adverse events

Overall there were 443 adverse events; 76/97 (78%) patients had one or more events; 47/97 (48%) patients had an adverse event in the first 6 months and 29/89 (33%) in the second 6 months (Table 4); 4/97 (4%) of patients had a serious adverse event, 3 in the first 6 months and 1 in the second 6 months; 220/443 (50%) of adverse events involved the musculoskeletal system and only 223/443 (50%) involved other systems. No relationships were identified between treatment tapering and adverse events.

**Table 4:** Serious and adverse events by body system and intervention group

Body system	Proof of principle phase (0–6 months)						Exploratory phase (6–12 months)							
	Tapered 33%, n = 15 (96 episodes)		Tapered 66%, n = 13 (71 episodes)		Controls, n = 19 (120 episodes)		Tapering 33% until stop, n = 9 (54 episodes)		Tapering 66% until stop, n = 4 (28 episodes)		Re-randomized control group to			
											Tapered 33%, n = 10 (43 episodes)		Tapered 66%, n = 6 (35 episodes)	
	Serious	All	Serious	All	Serious	All	Serious	All	Serious	All	Serious	All	Serious	All
Cardiovascular		3		1		3		0		0		2		0
Digestive		13		3		12		2		0		6		7
ENT		5		1		5		4		2		5		7
Endocrine/metabolic		0		0		0		0		0		0		0
Genitourinary	1	1		2		3		0		0		2		0
Haematological		0	1	1		6		1		0		0		0
Mental		0		0		1		0		2		0		5
Musculoskeletal		51		37		36		45		18		19		14
Nervous system		4		1		4		0		0		0		0
Ophthalmological		0		0		4		0		0		0		1
Respiratory		8		8		33		2		1		8		7
Skin	1	11		0		13		0		5		0	1	0

Seventy-six patients had an adverse event; of those, 47 patients had an AE in the first 6 months and 29 in the next 6 months.

## Discussion

OPTTIRA shows reducing TNF inhibitor doses by one-third in RA patients with stable low disease activity or remission also taking DMARDs has no impact on disease activity or the frequency of flares. However, the limited number of patients included in each arm restricts the importance of its finding for clinical practice. Most patients were in remission; there was some evidence patients with low disease activity scores had more flares. Greater reductions in TNF inhibitor doses led to more flares and higher disease activity levels but had no impact on disability. TNFi could be stopped in many patients without major negative impacts upon their disease.

The balance of evidence from OPTTIRA and previous trials and observational studies of tapering and stopping TNFi is that modest dose reduction is possible in RA patients with good responses to TNFi who remain on conventional DMARDs. Indeed there is little evidence favouring maintaining standard doses in such patients. OPTTIRA showed that reducing biologic doses by one-third had no impact on clinical, functional or health status assessments and did not result in more flares. TNFi are expensive with uncertainties about their overall cost-effectiveness [26, 27] and in patients with good responses tapering appears less expensive than maintaining full doses without major negative impacts.

The evidence favouring major reductions in TNF inhibitor dose, such as reducing by two-thirds, is less clear-cut. OPTTIRA, other trials and observational studies all found this gives more flares and worse clinical and health status. However, the negative impacts are modest and in some patients treatment can be stopped without obvious disadvantages. Considering stopping treatment may be reasonable in selected cases.

Reductions in TNF inhibitor doses in stable RA have not been approved by regulatory agencies and therefore organizations like the National Institute for Health and Clinical Excellence cannot recommend such approaches [28]. Nevertheless, individual clinicians may decide, based on overall assessments of risks and benefits, to offer dose reductions to some patients. The impact of tapering TNFi is broadly similar to reducing the doses of conventional DMARDs; reducing MTX doses can be achieved in some patients but stopping treatment increases flares. Defining patients likely to flare is an important future research goal.

Adverse events were common in OPTTIRA and often involved the musculoskeletal system. Only four were serious events and these were unrelated to tapering or stopping treatment. The serious adverse event rate was similar to that in the systematic review of tapering by van Herwaarden *et al.* [7] who reported 5% of patients had serious adverse events. Such adverse event rates are likely to be commonplace in treated RA patients.

TNFi are expensive, and while using effective biosimilars [29] will reduce costs, such biologic treatments will never become inexpensive. Consequently the clinical and the economic benefits of good responders remaining on standard doses of TNFi will diverge. As the disadvantages of tapering and trying to stop TNFi are relatively small, clinicians and healthcare funders may consider it preferable to explore reducing and stopping treatment when patients achieve good disease control. In early RA, using biologics initially may result in sustained biologic-free and drug-free remissions [30, 31]; OPTTIRA and similar trials have not evaluated this possibility. Nevertheless it may be important because achieving sustained deep remission may be of critical consequence in early disease.

Our trial had several strengths. It involved a range of RA patients across multiple English centres, making its findings likely to be generalizable. It evaluated two tapering regimens and stopping treatment, ensuring several strategies for reducing TNF inhibitor doses. It had several weaknesses. First, it was relatively small and enrolled only 97 patients. Its lack of power precludes robust conclusions in secondary analyses evaluating the impact of tapering on HAQ within the 66% tapering group. Secondly, it was relatively short and assessors were not blinded, in keeping with other pragmatic trials in the area. Thirdly, it did not look at all responders to TNFi, only those who had sustained low disease activity or remission; patients who almost achieved low disease activity may respond similarly to those enrolled in the trial. Fourthly, it only studied two TNFi, and may not apply to other TNFi. Fifthly, it did not consider sustained flares though there is some evidence [32] these are more important than transient flares, nor did it assess flares from patients’ perspectives, which recent research has highlighted as being relevant [33–35]. Sixthly, many patients did not wish to participate in OPTTIRA; our non-participation rate was substantially higher than some trials like that reported by Moghadam *et al.* [11] but lower than others [8]. Similar high non-participation rates have occurred in other RA trials from our unit [36, 37]. This difference may represent different ways of collecting data about screening or national differences in patients’ views on trials. However, caution is needed when considering our results’ generalizability. Finally, only limited information was available on radiological outcomes.

An issue in RA treatment, particularly relevant tapering biologics, is identifying patient sub-groups likely to respond well to the treatment strategy. Analysis of predictors in the BehandelStrategieën (BeSt) trial [38] showed Anti-citrullinated protein antibody (ACPA) was an important indicator of flares after stopping infliximab. The impact of ACPA status on response to tapering was also reported by Haschka *et al.* [10]. More recently Rech *et al.* [39] showed combining ACPA status with multibiomarker disease activity assessment predicted relapses in over 80% of patients. An alternative is using US assessments to predict response to tapering [40, 41]. There is evidence in our trial that tapering may be best in patients achieving deep remissions; further work is needed to clarify this possibility.

The most effective and cost-effective strategy for using biologics in RA patients remains uncertain. As current biologics are not curative in established RA patients, the rationale for their indefinite use in treatment responders is debatable. OPTTIRA shows modest tapering has no negative clinical impacts. We consider there is sufficient evidence for clinicians to reduce TNF inhibitor doses in some treatment responders. This approach may be more cost-effective than their continued use though it risks short-term disadvantages as flares are a burden to patients. Its impacts on disease status and healthcare costs need further evaluation, particularly as we found no evidence that tapering worsened patient-assessed outcomes.

## Supplementary Material

Supplementary Tables

Supplementary Figure S1
