# Supplementary material for: Optimizing treatment with tumour necrosis factor inhibitors in rheumatoid arthritis—a proof of principle and exploratory trial: is dose tapering practical in good responders?
Source: Rheumatology (Oxford). 2017 Aug 17;56(11):2004–14. doi: 10.1093/rheumatology/kex315 (PMC5722050; doi:10.1093/rheumatology/kex315)
Supplement: Supplementary Tables [file rhe-16-1831-file003_kex315.docx]

**SUPPLEMENTARY DATA**

**Supplementary Table S1: Etanercept Dosing Schedule Months 0-6 (mg)**

| *Week* | 1 | 2 | 3 | 4 | 5 | 6 | 7 | 8 | 9 | 10 | 11 | 12 | 13 | 14 | 15 | 16 | 17 | 18 | 19 | 20 | 21 | 22 | 23 | 24 | 25 | 26 |
| --- | --- | --- | --- | --- | --- | --- | --- | --- | --- | --- | --- | --- | --- | --- | --- | --- | --- | --- | --- | --- | --- | --- | --- | --- | --- | --- |
| *Group 1* | 0 | 50 | 50 | 50 | 0 | 50 | 50 | 50 | 0 | 50 | 50 | 50 | 0 | 50 | 50 | 0 | 50 | 50 | 0 | 50 | 50 | 0 | 50 | 50 | 0 | 50 |
| *Group 2* | 0 | 50 | 50 | 50 | 0 | 50 | 50 | 0 | 50 | 50 | 0 | 50 | 0 | 50 | 0 | 50 | 0 | 50 | 0 | 50 | 0 | 0 | 50 | 0 | 0 | 50 |
| *Control* | 50 | 50 | 50 | 50 | 50 | 50 | 50 | 50 | 50 | 50 | 50 | 50 | 50 | 50 | 50 | 50 | 50 | 50 | 50 | 50 | 50 | 50 | 50 | 50 | 50 | 50 |

**Supplementary Table S2. Adalimumab Dosing Schedule Months 0-6 (mg)**

| Week | 2 | 4 | 6 | 8 | 10 | 12 | 14 | 16 | 18 | 20 | 22 | 24 | 26 |
| --- | --- | --- | --- | --- | --- | --- | --- | --- | --- | --- | --- | --- | --- |
| *Group 1* | 0 | 40 | 40 | 40 | 0 | 40 | 40 | 0 | 40 | 40 | 0 | 40 | 40 |
| *Group 2* | 0 | 40 | 0 | 40 | 0 | 40 | 0 | 40 | 0 | 0 | 40 | 0 | 0 |
| *Control* | 40 | 40 | 40 | 40 | 40 | 40 | 40 | 40 | 40 | 40 | 40 | 40 | 40 |

**Supplementary Table S3: Etanercept Dosing Schedule Months 7-12 (mg)**

| *Week* | 27 | 28 | 29 | 30 | 31 | 32 | 33 | 34 | 35 | 36 | 37 | 38 | 39 | 40 | 41 | 42 | 43 | 44 | 45 | 46 | 47 | 48 | 49 | 50 | 51 | 52 |
| --- | --- | --- | --- | --- | --- | --- | --- | --- | --- | --- | --- | --- | --- | --- | --- | --- | --- | --- | --- | --- | --- | --- | --- | --- | --- | --- |
| *Group 1* | 0 | 50 | 0 | 0 | 50 | 0 | 0 | 0 | 50 | 0 | 0 | 0 | 0 | 0 | 0 | 0 | 0 | 0 | 0 | 0 | 0 | 0 | 0 | 0 | 0 | 0 |
| *Group 2* | 0 | 0 | 0 | 50 | 0 | 0 | 0 | 0 | 50 | 0 | 0 | 0 | 0 | 0 | 0 | 0 | 0 | 0 | 0 | 0 | 0 | 0 | 0 | 0 | 0 | 0 |
| *Control A* | 0 | 50 | 50 | 50 | 0 | 50 | 50 | 50 | 0 | 50 | 50 | 50 | 0 | 50 | 50 | 0 | 50 | 50 | 0 | 50 | 50 | 0 | 50 | 50 | 0 | 50 |
| *Control B* | 0 | 50 | 50 | 50 | 0 | 50 | 50 | 0 | 50 | 50 | 0 | 50 | 0 | 50 | 0 | 50 | 0 | 50 | 0 | 50 | 0 | 0 | 50 | 0 | 0 | 50 |

**Supplementary Table S4: Adalimumab Dosing Schedule Months 7-12 (mg)**

| *Week* | 28 | 30 | 32 | 34 | 36 | 38 | 40 | 42 | 44 | 46 | 48 | 50 | 52 |
| --- | --- | --- | --- | --- | --- | --- | --- | --- | --- | --- | --- | --- | --- |
| *Group 1* | 0 | 0 | 40 | 0 | 0 | 0 | 0 | 0 | 0 | 0 | 0 | 0 | 0 |
| *Group 2* | 40 | 0 | 0 | 40 | 0 | 0 | 0 | 0 | 0 | 0 | 0 | 0 | 0 |
| *Control A* | 0 | 40 | 40 | 40 | 0 | 40 | 40 | 0 | 40 | 40 | 0 | 40 | 40 |
| *Control B* | 0 | 40 | 0 | 40 | 0 | 40 | 0 | 40 | 0 | 0 | 40 | 0 | 0 |

**Supplementary Table S5: Individual mean (standard errors) for secondary outcome measures by treatment group**

| **Outcome** | **33% Tapering (n=26)** | | | **66% Tapering (n=21)** | | | **Controls (n=50)** | | |
| --- | --- | --- | --- | --- | --- | --- | --- | --- | --- |
|  | *Baseline* | *6 months* | *Change* | *Baseline* | *6 months* | *Change* | *Baseline* | *6 months* | *Change* |
|  | *Mean (SE)* | *Mean (SE)* | *Mean (SE)* | *Mean (SE)* | *Mean (SE)* | *Mean (SE)* | *Mean (SE)* | *Mean (SE)* | *Mean (SE)* |
| DAS28-ESR | 1.75 (0.16) | 1.97 (0.13) | 0.22 (0.17) | 1.74 (0.20) | 2.28 (0.26) | 0.54 (0.20) | 1.96 (0.11) | 2.16 (0.13) | 0.21 (0.11) |
| DAS28-CRP | 2.14 (0.09) | 2.15 (0.09) | 0.01 (0.13) | 2.02 (0.11) | 2.29 (0.22) | 0.27 (0.17) | 2.13 (0.08) | 2.30 (0.10) | 0.17 (0.08) |
| Tender Joint Counts | 0.27 (0.13) | 0.36 (0.15) | 0.16 (0.19) | 0.52 (0.28) | 2.05 (0.81) | 1.55 (0.78) | 0.49 (0.13) | 0.96 (0.25) | 0.47 (0.22) |
| Swollen Joint Counts | 0.35 (0.17) | 0.36 (0.14) | 0.01 (0.16) | 0.05 (0.05) | 0.65 (0.27) | 0.60 (0.29) | 0.42 (0.16) | 0.57 (0.22) | 0.14 (0.18) |
| ESR | 14.5 (3.15) | 13.16 (2.29) | -1.32 (2.83) | 13.45 (3.28) | 11.75 (2.30) | -1.70 (2.15) | 13.71 (1.71) | 13.76 (1.55) | 0.04 (1.10) |
| CRP | 5.38 (0.82) | 4.56 (0.62) | -0.84 (0.97) | 6.90 (1.92) | 3.95 (1.05) | -2.95 (1.88) | 5.67 (0.97) | 6.90 (1.82) | 1.22 (1.03) |
| Assessor Global | 5.92 (1.65) | 7.08 (1.63) | 1.16 (1.12) | 6.15 (1.48) | 12.85 (3.55) | 6.70 (3.11) | 5.90 (1.01) | 9.39 (2.10) | 3.49 (2.10) |
| Patient Global | 10.4 (2.16) | 10.80 (2.14) | 0.40 (1.25) | 10.35 (2.74) | 14.70 (3.30) | 4.35 (2.86) | 9.67 (1.56) | 11.90 (2.15) | 2.22 (2.08) |
| HAQ | 0.80 (0.15) | 0.80 (0.16) | 0 | 0.60 (0.17) | 0.69 (0.17) | 0.09 (0.07) | 0.81 (0.11) | 0.73 (0.11) | -0.07 (0.05) |
| EQ5D-3L | 0.82 (0.04) | 0.75 (0.05) | -0.07 (0.04) | 0.82 (0.03) | 0.77 (0.05) | -0.04 (0.04) | 0.77 (0.03) | 0.77 (0.03) | -0.01 (0.03) |
| VAS Pain | 9.7 (2.6) | 17.2 (4.0) | 1.1 (2.2) | 12.1 (2.9) | 18.6 (4.5) | 2.7 (2.6) | 12.6 (2.2) | 14.4 (2.7) | -0.7 (2.2) |
| FACIT Fatigue | 38.9 (1.5) | 38.7 (1.8) | -0.2 (1.0) | 38.1 (2.2) | 39.6 (2.4) | 1.5 (1.8) | 40.0 (1.1) | 39.3 (1.3) | -0.7 (0.8) |
| Larsen Score | 48.1 (9.4) | 49.2 (9.6) | 1.1 (0.6) | 49.4 (9.3) | 51.1 (9.4) | 1.9 (0.7) | 66.9 (7.2) | 68.2 (7.3) | 1.3 (0.5) |
