# Supplementary figures and images for: Optimizing treatment with tumour necrosis factor inhibitors in rheumatoid arthritis—a proof of principle and exploratory trial: is dose tapering practical in good responders?
Source: Rheumatology (Oxford). 2017 Aug 17;56(11):2004–14. doi: 10.1093/rheumatology/kex315 (PMC5722050; doi:10.1093/rheumatology/kex315)

Supplementary Figure S1. Consort flowchart for trial

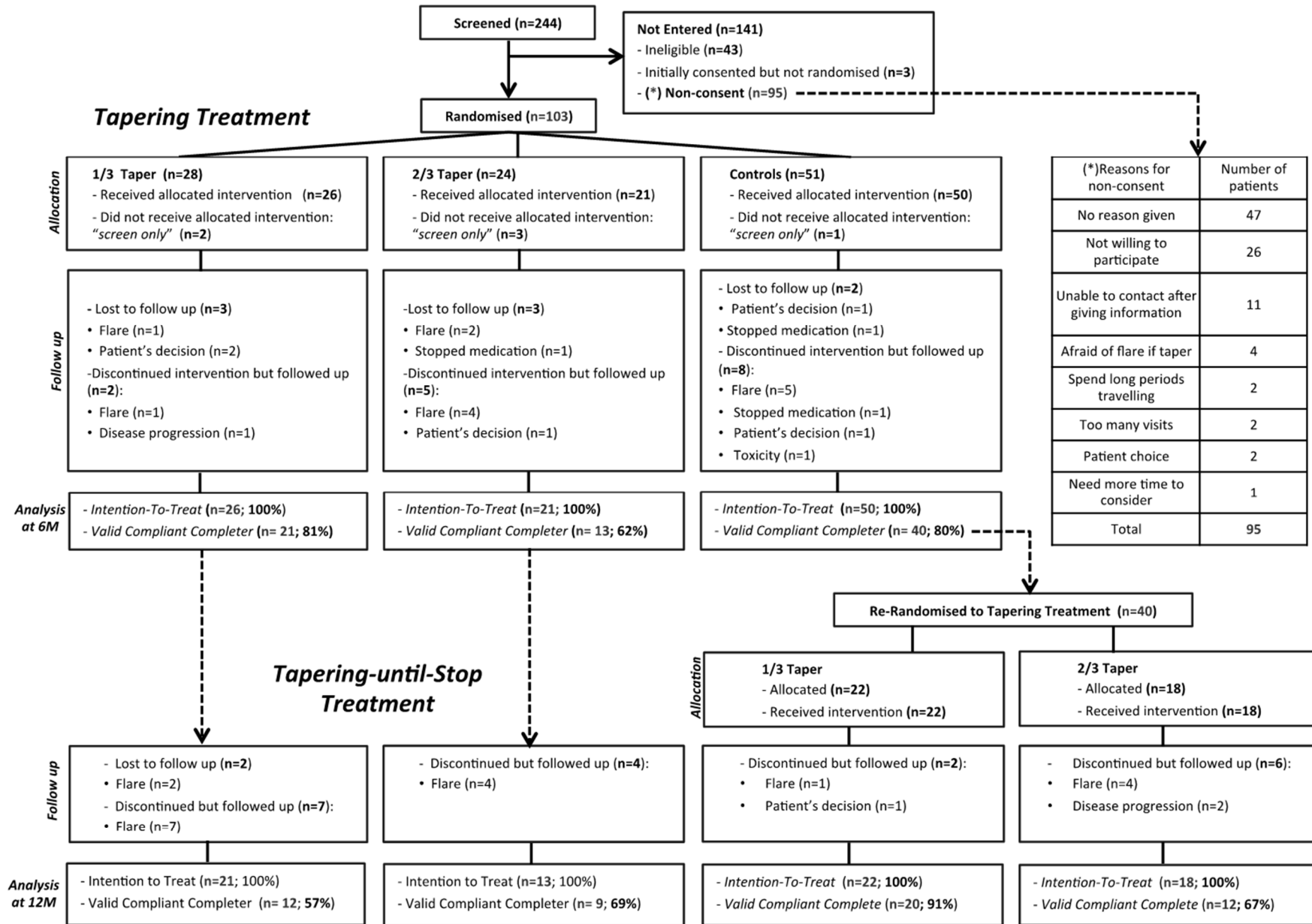

Supplement: Supplementary Figure S1 [file rhe-16-1831-file004_kex315.pdf]
